# Simultaneous Identification and Dynamic Analysis of Saccharides during Steam Processing of Rhizomes of *Polygonatum cyrtonema* by HPLC–QTOF–MS/MS

**DOI:** 10.3390/molecules23112855

**Published:** 2018-11-02

**Authors:** Jian Jin, Jia Lao, Rongrong Zhou, Wei He, You Qin, Can Zhong, Jing Xie, Hao Liu, Dan Wan, Shuihan Zhang, Yuhui Qin

**Affiliations:** 1 Institute of Chinese Materia Medica, Hunan Academy of Chinese Medicine, Changsha 410013, China; jinjian2016@163.com (J.J.); 20162010@stu.hnucm.edu.cn (Y.Q.); canzhong651@163.com (C.Z.); axxj2057@163.com (J.X.); 350013@hnucm.edu.cn (H.L.); 350017@hnucm.edu.cn (D.W.); 2 School of Chinese Medicine, Hunan University of Chinese Medicine, Changsha 410208, China; 3 2011 Collaboration and Innovation Center for Digital Chinese Medicine in Hunan, Changsha 410208, China; 4 Resgreen Group International Inc., Changsha 410329, China; laojia1973@163.com (J.L.); hewei3218@126.com (W.H.); 5 College of Pharmacy, Changchun University of Chinese Medicine, Changchun 130117, China; rz172@georgetown.edu

**Keywords:** *Polygonatum cyrtonema*, saccharides, oligosaccharides, fructose, HPLC–QTOF–MS/MS, steaming

## Abstract

The sweet rhizomes of *Polygonatum cyrtonema* are widely used as a tonic and functional food. A sensitive and rapid analytical method was developed for simultaneous identification and dynamic analysis of saccharides during steam processing in *P. cyrtonema* using HPLC–QTOF–MS/MS. Fructose, sorbitol, glucose, galactose, sucrose, and 1-kestose were identified, as well as a large number of oligosaccharides constituted of fructose units through β-(2→1) or β-(2→6). Polysaccharides and oligosaccharides were decomposed to monosaccharides during a steaming process, since the contents of glucose, galactose, and fructose were increased, while those of sucrose, 1-kestose, and polysaccharides were decreased. The high content of fructose was revealed to be the main determinant for increasing the level of sweetness after steaming. The samples of different repeated steaming times were shown to be well grouped and gradually shift along the PC1 (72.4%) axis by principal component analysis. The small-molecule saccharides, especially fructose, could be considered as markers for the steaming process of rhizomes of *P. cyrtonema*.

## 1. Introduction

The rhizomes of *Polygonatum cyrtonema* are wildly used as a tonic and functional food in China. The use of this plant is documented in the well-known ancient Pharmacopoeia “*Shennong Bencao Jing*”, and it is considered as a “Top grade” herb. Its main efficacy includes replenishing energy, strengthening immunity, and treating fatigue, weakness, loss of appetite, and so on. The genus *Polygonatum* has also been used in some other regions, such as India, Japan, Europe, and North America [1].

There are various ways to cook the rhizomes of *Polygonatum*. They are often cooked with meats or porridges, made into tea or medicated wine, or consumed as fruits or vegetables [2]. It is worth noting that raw rhizomes without processing treatment are rarely directly used. Raw rhizomes are often processed to enhance their tonic function by repeated steaming and drying. In particular, according to its traditional uses, the rhizomes of *Polygonatum* are processed by steaming nine times, which is recently combined with autoclave as an efficient approach. Traditional quality control is performed by qualitative index, described as changes such as rhizomes becoming black, soft, and especially, gaining a sweet taste. However, the changes of the chemical composition during the steaming process are not understood.

With respect to chemical composition, there could be steroidal saponins, triterpenoid saponins, homoisoflavanones, polysaccharides, and lectins in the *Polygonatum* plants [1]. Polysaccharides are the main chemical components, and possess various pharmacological actions, such as antioxidant protection and immune-regulation [3]. The quality of *P. cyrtonema* is evaluated depending on the content of polysaccharides, with a minimal content of 7% required by Chinese Pharmacopoeia. Polysaccharides from *Polygonatum* plants are composed of different ratios of monosaccharides, mainly including mannose, galactose, glucose, fructose, rhamnose, arabinose, and galacturonic acid [1]. A branched fructan was also isolated from *P. cyrtonema* [4]. A large number of monosaccharides, oligosaccharides, and polysaccharides were found in rhizomes of *Polygonatum* plants, making it worthwhile to try to evaluate the processed products of rhizomes of *P. cyrtonema* with the help of saccharides in order to clarify the reason why the processed rhizomes of *Polygonatum* gradually take on a sweet taste.

Commonly-used analytical modes of saccharides include nuclear magnetic resonance (NMR) [5], gas chromatography (GC) [6], and liquid chromatography (LC) [7,8]. LC is widely used thanks to its various detectors, while NMR cannot be used to detect trace amounts of saccharides, and GC requires tedious derivatization [9]. However, because the absence of chromophore and fluorophore groups, avoiding direct detection by ultraviolet and fluorescence or diode array detectors, pre-column derivatization is often employed in LC, which is time consuming and could introduce extra variations into the final data [10]. LC equipped with an evaporative light scattering detector (ELSD) could be used to detect the monosaccharides; for instance, glucose, fructose, and sucrose were examined in the raw materials of *agave* leaf [11]. Structural information can even be provided by well-developed analytical techniques; for example, mass spectrometry (MS) coupled with LC is currently a more powerful analytical tool for saccharide analysis [10]. Sucrose, fructose, and glucose in plant tissues can be unambiguously determined by LC–MS [10]. Three major tri–saccharides in wheat flour were also determined by LC–MS [12]. It is worth mentioning that most previous study focused on fructose, glucose, sucrose and other oligosaccharides, but seldom distinguished glucose and galactose, which are the most common isomeric hexoses present in physiology. LC coupled with atmospheric pressure chemical ionization (APCI)–MS was developed to identify most of the standard saccharides, but it is still hard to separate glucose and galactose [13]. It seems that no simple method is available for the differentiation of these two isomers, since current methods required either derivatization techniques based on the reaction of reducing sugars with 1-phenyl-3-methyl-5-pyrazolone, phenylhydrazones [14], or the help of a zinc diethylenetriamine (Zndien) metal-ligand system [15].

In *Polygonatum* plants, polyphenolic antioxidants have ever been characterized by high-performance liquid chromatography (HPLC)–quadrupole-time-of-flight (QTOF)–MS, a useful technology for structural identification, even when standard compounds are not available [16]. Nevertheless, few studies of simultaneous analysis of saccharides in rhizomes of *Polygonatum* were reported, let alone characterization of the changes of the saccharide composition during the nine times of repeated steaming process. In the present study, an efficient and sensitive method was developed by HPLC–QTOF–MS/MS to profile and identify saccharides, including glucose and galactose, in fresh and processed rhizomes of *P. cyrtonema* by nine-time steaming. The present study will provide not only a useful tool for the analysis of saccharides, but also information for the improvement of *Polygonatum* steam process.

## 2. Results and Discussion

### 2.1. Appearance Change

**Figure 1:**
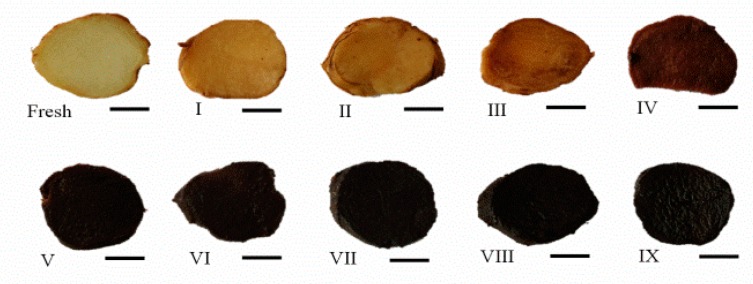
Samples of the rhizomes of *P. cyrtonema* are processed by different times of repeated steaming; “fresh” implies without steam process; roman numerals indicate the number of times of steaming; bar, 1 cm.

The rhizomes of *P. cyrtonema* were processed by repeated steaming for one to nine times, shown in Figure 1.

Steaming treatment is a usual food processing technology for the pretreatment of lignocellulosic materials [17]. In recent decades, an autoclaving steam process has emerged as an industrially-adopted method, not only to deliver a wide range of high quality products to meet the traditional Chinese requirements, but also to extend shelf life as a technique of food pasteurization to kill food pathogens and ensure food safety. Heavy metals in *Polygonatum* roots, such as Pb and Cd, could also be eliminated by exposure to high temperature, pressurized steaming conditions [18]. The well-known medicinal herb black ginseng is also usually treated with steaming; ginsenoside composition changes during the steaming process [19,20]. Traditionally, rhizomes of *Polygonatum* were used mainly as a crude drug, while in recent years, they are more popular as food when cooked with meats or porridges, made into tea or medicated wine, or consumed as fruits or vegetables [2]. The color and taste are two of the most crucial sensory properties of food. From fresh to processed samples with different numbers of repeated steaming, the samples changed to black, and became sweeter. But after steaming four times, the color appeared stable. The Maillard reaction, during which sugars react with amino acids under thermal conditions [21], would be largely responsible for the dark-colored appearance of processed rhizomes of *P. cyrtonema*. For the sweet taste, the present study was designed to systematically assess the saccharides, especially the small-molecular saccharides, between fresh and processed rhizomes of *P. cyrtonema*.

### 2.2. Optimization and Validation of the HPLC–QTOF–MS/MS Method

**Figure 2:**
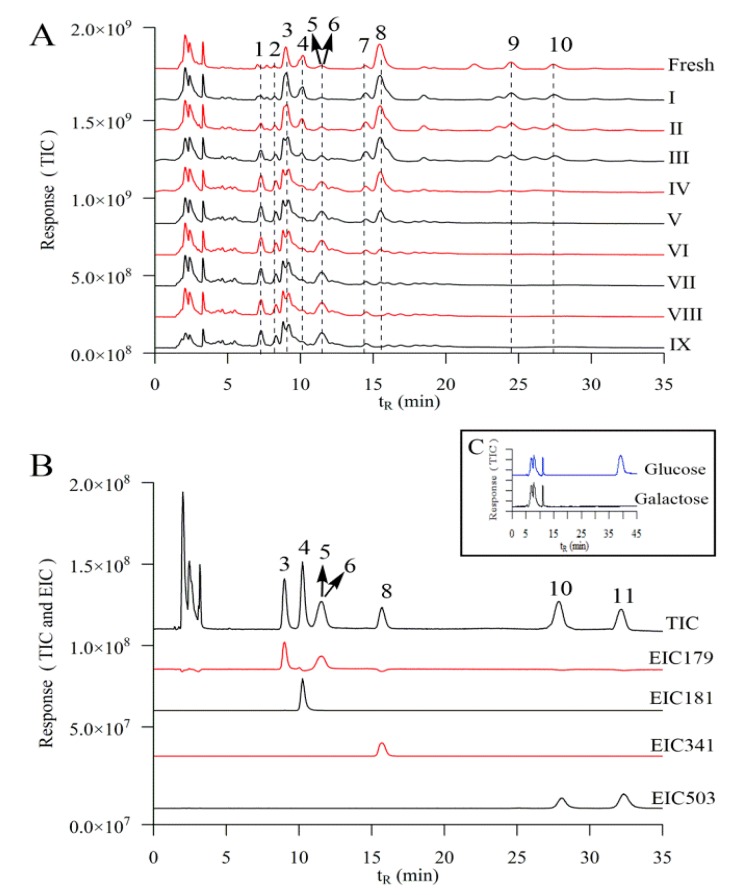
Chromatogram of the extract of the rhizomes of *P. cyrtonema* are processed by different times of repeated steaming (**A**), standards of small–molecular saccharides (**B**) by HPLC–QTOF–MS/MS at solvent flow rate 1.0 mL/min, glucose and galactose at solvent flow rate 0.3 mL/min (**C**); 1, fructose derivative; 2, glucose derivative; 3, fructose; 4, sorbitol; 5, glucose; 6, galactose; 7, hexose; 8, sucrose; 9, trisaccharide; 10, 1-kestose; 11, raffinose; TIC, total ion chromatogram; EIC, extracted ion chromatogram; “fresh” means without steaming; roman numerals indicate the number of times of steaming.

For simultaneous determination of saccharides and their derivatives, the optimal chromatographic conditions were investigated. Various mixtures of water, methanol, and acetonitrile at flow rate 1.2 mL/min, 1.0 mL/min, 0.8 mL/min, 0.5 mL/min and 0.3 mL/min were tested as the mobile phase. The detection temperature was tested at 15, 25, 35, and 45 °C. Eventually, the optimal chromatographic conditions were as follows: Prevail Carbohydrate column, with acetonitrile 75% and ultrapure water 25% as mobile phase at 1.0 mL/min at 25 °C. As shown in Figure 2A, the peaks in fresh and processed samples are separated by HPLC–QTOF–MS/MS. This condition was suitable for most of the small molecular standards (Figure 2B), except for glucose and galactose, which appeared at the same retention time 11.60 min. Surprisingly, when mobile phase flow rate decreased to 0.3 mL/min, only the standard glucose appeared at retention time 39.56 min, while no peak of the galactose appeared using sole standard solutions (Figure 2C). Then, the quantitative determination of glucose in samples was carried out with the mobile phase flow rate at 0.3 mL/min for the peak at retention time 39.56 min. The content of galactose was calculated by subtracting the glucose from the peak at retention time 11.60 min with mobile phase flow rate at 1.0 mL/min.

Noting that the peak of TIC and EIC will be saturated and even lead to bifurcation when the standard concentration is too high (Figure S1), the standard stock solutions containing fructose, glucose, galactose, sucrose and 1-kestose were prepared and diluted to a series of appropriate concentrations for the construction of calibration curves. These curves showed good linearity, and the correlation coefficients were found to be in the range with R^2^ 0.99 for all of the compounds in a certain concentration range (Table 1), consistent with saccharides determined by hydrophilic interaction liquid chromatography (HILIC)–TOF–MS technology [7]. The limits of detection (LOD) and limits of quantification (LOQ) were in the range of 0.41–3.63 μg/mL and 1.33–12.10 μg/mL, respectively. The intraday and interday variations of the analytes (RSDs) were within 0.93–1.91% and 1.82–3.51%, respectively. The RSDs for stability were lower than 3.01%. The average recoveries of the standard compounds ranged from 94.3% to 107.5%, and their RSDs were within 6.39% (Table 2). All these results demonstrated that the developed quantitative HPLC–QTOF–MS method was linear, precise, stable, sensitive, and accurate enough for the determination of small-molecular saccharides in rhizomes of *P. cyrtonema*. There are studies using HPLC system coupled with MS to investigate saccharides in plant tissues or products, but previous reports mainly investigated glucose, fructose and sucrose [10,22]. LC–APCI–MS was developed to distinguish saccharides with different types of columns, but it is still hard to separate glucose and galactose [13]. To the best of our knowledge, this is the first time that small-molecule saccharides, including glucose and galactose, were separated and determined by the highly sensitive and rapid HPLC–QTOF–MS/MS method using a common column without derivatization.

### 2.3. Identification of Small-Molecule Saccharides

Since the chromatographic peaks could not be identified unambiguously by HPLC retention time alone, HPLC–QTOF–MS/MS was used to confirm identities by comparing the retention time and molecular ions for each peak. In this experiment, the mass spectral conditions were optimized in negative-ion mode, and the six compounds exhibiting distinct quasi-molecular ions [M − H]^−^ and [M + COOH]^−^ were optimized in this mode: fructose (peak 3), molecular weight (MW) 180 *m*/*z* = 179 [M − H]^−^, sorbitol (peak 4) (MW 182) *m*/*z* = 181 [M − H]^−^, glucose (peak 5) (MW 180) *m*/*z* = 179 [M − H]^−^, galactose (peak 6) (MW 180) *m*/*z* = 179 [M − H]^−^, sucrose (peak 8) (MW 342) *m*/*z* = 341 [M − H]^−^ and *m*/*z* = 387 [M + COOH]^−^, 1-kestose (peak 10) (MW 504) *m*/*z* = 503 [M − H]^−^ and *m*/*z* = 549 [M + COOH]^−^ and raffinose (peak 11) (MW 504) *m*/*z* = 503 [M − H]^−^ and *m*/*z* = 549 [M + COOH]^−^. For the monosaccharide, the fragment ions demonstrated the same *m*/*z* value at 179, 161, 143, 113 and 101. For the disaccharide sucrose, the fragment ions are at *m*/*z* 341, 179, 161, 143, 119 and 101. For the trisaccharides, i.e., 1-kestose and raffinose, the fragment ions are at *m*/*z* 503, 341, 323, 221, 179, 161, 143, 113 and 101 (Table 3).

In the HPLC–QTOF–MS/MS spectra, the molecular ions of each compound agreed well with the chemical structures. Basing on the above results, the standard compounds of fructose, sorbitol, glucose, galactose, sucrose, and 1-kestose were identified in the extract of the rhizomes of *P. cyrtonema*, and the identities of each peak were clearly confirmed. However, raffinose, a common oligosaccharide in food [23], was not found in fresh or processed samples.

**Figure 3:**
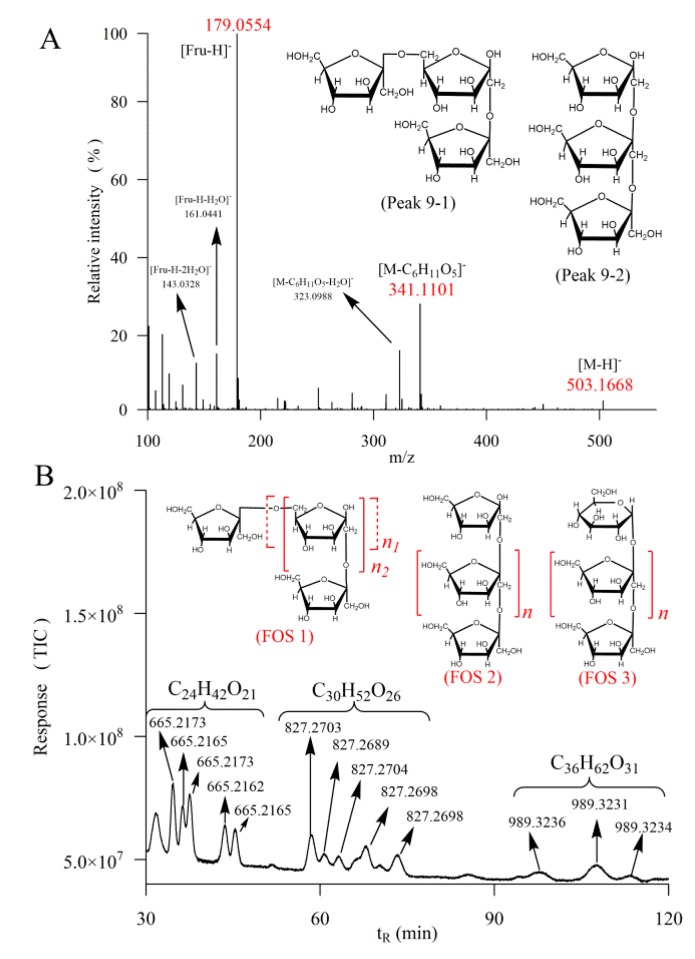
MS/MS spectrum, the two speculated structures of peak 9 (**A**) and LC spectrum with *m*/*z* values, speculated molecule formulas and structures in fresh rhizomes of *P. cyrtonema* by HPLC–QTOF–MS/MS with mobile phase flow rate at 1.2 mL/min (**B**). FOS, fructo-oligosaccharides.

It is worth noting that there were also obvious peaks 1, 2, 7, and 9 in the rhizomes of *P. cyrtonema*. Peak 1 and 2 were shown to be due to the same fragment ions to monosaccharide with *m*/*z* value at 179, 161, 143, 113, and 101, indicating a component of hexose. The fragment ion at *m*/*z* 323 could be due to [341 − H_2_O]^−^, where 341 is the characterized ion of disaccharide. Then, peak 1 and 2 could be hexose derivatives formed by dehydration from disaccharides. The *m*/*z* values of peak 7 fragment ions were at 179, 161, 143, 113, and 101, the same with that of monosaccharide, indicating a hexose structure. Peak 9 demonstrated the same fragment ions with those of 1-kestose and raffinose, with *m*/*z* value at 503, 341, 323, 221, 179, 161, 143, 113, and 101, also indicating a trisaccharide. 1-kestose is formed of two molecules of fructose linked by β-(2→1) d-fructosyl-fructose bonds, and terminated with one glucose unit [24]. Noting that for the retention time, the peak of fructose is 2.48 min before glucose, peak 9 could be a trisaccharide composed of three molecules of fructose, because the retention time of peak 9 was 2.78 min before that of 1-kestose. There could be two possibilities for the linkage of this identified trisaccharide. One is that the molecules of fructose are linked by β-(2→1) and β-(2→6) as mixed type F3 fructan in inulin [25], namely MF3 as is shown in Figure 3A peak 9-1. The other could be simpler that a molecule of fructose replaces the glucose in 1-kestose, keeping the straightforward structure, namely SF3 (Figure 3A peak 9-2).

In order to further characterize the fructo-oligosaccharides (FOS), the mobile phase increased to 1.2 mL/min for 120 min to overview the outcome. It could be speculated that the peaks appearing at *m*/*z* value 665, 827, and 989 could have molecular formulas C_24_H_42_O_21_ (tetrasaccharide), C_30_H_52_O_26_ (pentasaccharide) and C_36_H_62_O_31_ (hexasaccharide) (Figure 3B). These FOS should be derived from the trisaccharides, i.e., 1-kestose, MF3 and SF3, by lengthening the chains with adding a variable number of fructose units through β-(2→1) or β-(2→6), as is shown in Figure 3B FOS 1, FOS 2 and FOS 3. Fibers of *P. odoratum* have been found to be composed of arabinose, xylose, sorbose, mannose and galactose [26] and lectin in *P. cyrtonema* could be binding to mannose [27]. Another monosaccharide analysis study showed that all the complete hydrolytic polysaccharides of *P. cyrtonema* are determined to be heteropolysaccharides containing rhamnose, arabinose, xylose, mannose, galactose and glucose [28]. However, the present investigation found that oligosaccharides in *P. cyrtonema* were mainly made up of fructose and glucose. The speculated structures of FOS in the present work are consistent with those of the previous study, in which the neutral polysaccharide in *P. cyrtonema* was found to be a branched fructan, composed of (2→6)-linked β-d-fructofuranosyl residues and (2→1)-linked β-d-fructofuranosyl residues in the backbone [4]. Since the intake of prebiotic fructans could sustain health and overall well-being [29], the observation of a large number of FOS in *P. cyrtonema* could be an evidence to explain its functional benefits.

### 2.4. Dynamic Change of Saccharides

**Figure 4:**
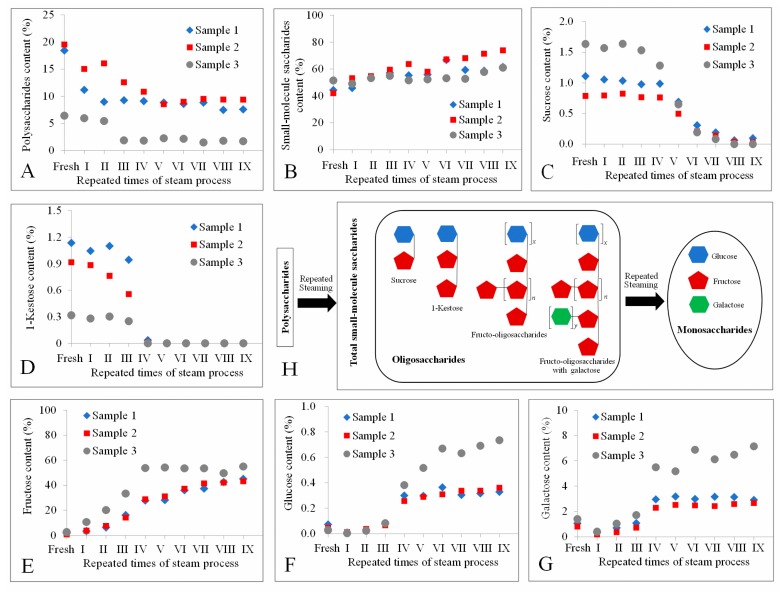
The content of polysaccharides (**A**), total small–molecule saccharides (**B**), sucrose (**C**), 1-kestose (**D**), fructose (**E**), glucose (**F**), galactose (**G**), in the rhizomes of *P. cyrtonema* processed by different times of repeated steaming and the proposed model of saccharides change during repeated steaming (**H**); “fresh” means without steam process; roman numerals indicate the number of times of steaming.

The contents of fructose, glucose, galactose, sucrose, and 1-kestose in three samples treated with different times of individual processing steps were measured by the developed method, as well as the total small-molecule saccharides and polysaccharides. Even though the saccharides in different *Polygonatum* samples usually varied [30], the present results demonstrated that there was an obvious influence on the quantities of small-molecule saccharides between fresh and steam processed samples. The dynamic changes and proposed model are illustrated in Figure 4.

The content of polysaccharides decreased, while the total small-molecule saccharides increased, indicating the polysaccharides were decomposed to small-molecule saccharides with the repeated times of steam process (Figure 4A,B). The measured oligosaccharides sucrose and 1-kestose were broke down forming glucose and fructose, because sucrose and 1-kestose were decreased (Figure 4C,D), whereas glucose and fructose increased (Figure 4E,F). Unexpectedly, the content of galactose also increased (Figure 4G), indicating this monosaccharide could be a branch linked to FOS in *P. cyrtonema*, as proposed in the model Figure 4H. Interestingly, steaming four times seems to be a landmark for these small-molecule saccharides, since their content appeared to be relatively stable after that. All these dynamic changes of polysaccharides and small-molecule saccharides caused by the decomposition of the glycosidic linkages, which could be broken by steam processing [26], resulting in an increase in the contents of glucose, galactose, and fructose, and a decrease in oligosaccharide and polysaccharides. It is reported that the fructose in Radix Rehmanniae gradually decreased, and the glucose remained relatively stable after steaming [9]. These could be species dependent, since the glycosidic linkages might be different in different kinds of plants.

Surprisingly, the content of fructose gradually increased to an extremely high value of about 50% of dry sample weight, while that of most food is less than 10% [31]. Only in a few foods, for instance honey, fructose, and glucose together account for 85–95%, and fructose usually predominates, which is responsible for the sweetness [32]. The change of fructose could be the main reason why the taste of the rhizomes of *P. cyrtonema* became sweeter after the steaming processing, since fructose is a major determinant of sweetness in food [10]. The high content of fructose is also in accordance with our speculation that oligosaccharides were mainly composed of fructose. It is reported that fructans isolated from the rhizomes of *P. odoratum* could be composed of 29 units of fructose and 1 unit of glucose [33]. A neutral polysaccharide with an average degree of polymerization of 28 from *P. cyrtonema* Hua is also mainly made up of fructose [4]. Therefore, it could be concluded that the high concentration of fructose probably came from the decomposition of fructo-oligosaccharides and polysaccharides.

### 2.5. PCA Statistical Analysis

**Figure 5:**
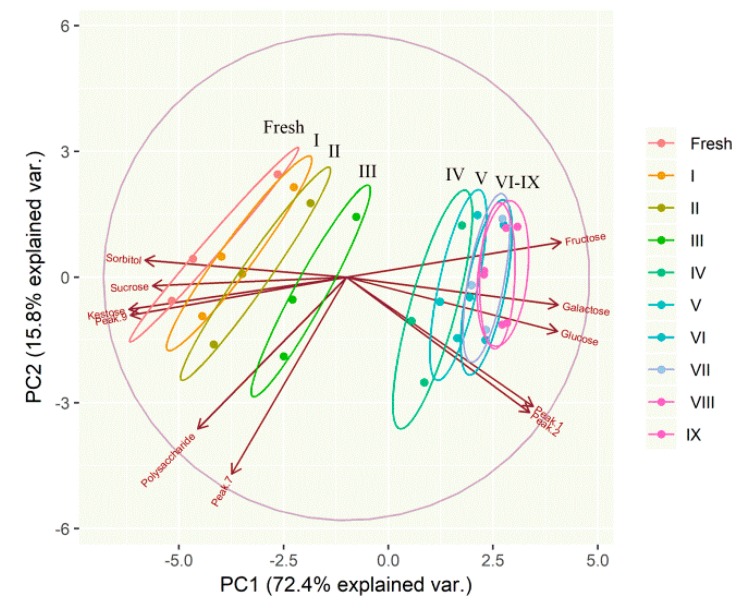
A biplot showing the samples of fresh and processed rhizomes of *P. cyrtonema* by different times of repeated steaming on a two-dimensional space derived from principal component analysis basing on identified saccharides and their derivatives; the arrows indicated the projections of the original features onto the principal components; “fresh” means without steam process; roman numerals indicate the number of times of steaming.

To overview the difference between fresh and processed rhizomes of *P. cyrtonema*, unsupervised PCA was performed. The contents of monosaccharides, oligosaccharides, identified saccharides derivatives, as well as polysaccharides, in all the samples of fresh and processed rhizomes were considered as the variables of PCA. The PCA biplot displayed the scores and loadings of the first two components (PC1 and PC2), revealing the projection of an observation on the subspace with score points (Figure 5). 72.4% and 15.8% of the variation in the pattern of concentrations of identified saccharides and derivatives were explained by PC1 and PC2, respectively. These two components together explained 88.2% of the variation.

The variables of fructose, glucose, galactose, peak 1, and peak 2 shown in the same direction correlated along PC1, whereas sucrose, 1-kestose, sorbitol and peak 9 were oppositely correlated to PC1. Polysaccharides and peak 7 were primarily correlated with PC2. The samples gradually shifted along PC1 axis with the increasing times of repeated steaming. PCA results could substantially separate fresh and processed rhizomes by 1–4 times of repeated steaming, but failed to distinguish samples processed with 4–9 times steaming, as there were a large number of samples overlapping between these groups. This result was in consistent with the previous observation that the color and aforementioned compounds were relatively stable after steaming four times. To a certain degree, present PCA study was in accordance with previous report that the raw and steamed samples were separated into two groups by PCA in potato strips [34] and in Radix Rehmanniae samples [7,9]. To date, no compound has been used as a marker compound for processed *P. cyrtonema*. Then, these small–molecule saccharides might be considered as marker compounds. In particular, fructose, due to its high content, could be given special consideration as an important marker compound.

## 3. Materials and Methods

### 3.1. Chemicals and Reagents

Galactose (CAS 387116-33-2), 1-kestose (CAS 470-69-9) and d-raffinose (CAS 512-69-6) were obtained from National Institutes for Food and Drug Control (Beijing, China). d-fructose (CAS 57-48-7), d-glucose (CAS 50-99-7), d-sorbitol (CAS 50-70-4), and sucrose (CAS 57-50-1) were purchased from Hefei Bomei Biotechnology. Co., Ltd. (Hefei, China). Acetonitrile (HPLC–grade) was purchased from Merck (Darmstadt, Germany). Ultrapure water was purchased from Wahaha (Changsha, China). Other reagents were of analytical grade.

### 3.2. Preparation of Samples

Fresh rhizomes of *P. cyrtonema* were collected from Xinhua County, Hunan Province, China and authenticated by Professor Zhaoming Xie and Hao Liu from Hunan Academy of Chinese Medicine. The voucher specimens were kept in our department for future reference. Rhizomes of *P. cyrtonema* were cleaned, dried and then cut into thin slices (3 mm ± 1 mm) right after the harvest. The rhizomes in a glass bottle were processed by nine times repeated steaming with autoclave (121 °C, 0.12 MPa, 30 min); then, samples were kept in the autoclave until cooled to room temperature. Samples were collected after each cycle of steaming process.

### 3.3. Extraction of Saccharides

The steaming-processed rhizomes of *P. cyrtonema* were dried at 60 °C to a constant weight. Then, samples were ground by passing a 40-mesh screen and extracted by distilled water with ultrasonication (KM-500DB, 40 KHz, Kunshan Meimei Ultrasonic Instrument Co., Ltd., Jiangsu, China) for 30 min followed by maintenance at 90 °C for 60 min. After that, the extracts were centrifuged (3000× *g* for 15 min) to separate the supernatant from residues. Then, 10 mL of ethanol (95%) was added to 2 mL of the supernatant for precipitating the polysaccharides. The small-molecule saccharides, including monosaccharides and oligosaccharides, which were easier to dissolve in ethanol, were still dissolved in upper layer [35]. Then, the polysaccharides and small-molecule saccharides were separated by centrifugation (3000× *g*, 10 min). The precipitated polysaccharides were redissolved in distilled water for colorimetric quantitative measurement. The up layer was collected for colorimetric quantitative measurement of the total small-molecule saccharides and HPLC–QTOF–MS/MS analysis to identify monosaccharides and oligosaccharide compounds.

### 3.4. Spectrophotometric Quantitative Measurement

The concentration of total small-molecule saccharides and polysaccharides were determined with the phenol/sulfuric acid colorimetric method [36]. Briefly, 2 mL sample solution and 1 mL of 5% aqueous solution of phenol were mixed in a test tube. Subsequently, 5 mL of concentrated sulfuric acid was added rapidly to the mixture. Test tubes were shaken in an ultrasonic bath for 10 min and then left at room temperature for 20 min for color development. The absorbance of the acquired solution was measured at 490 nm on a UV–Vis spectrophotometer (UV 2450, Shimadzu Corporation, Kyoto, Japan). A reference solution was prepared in an identical manner as that explained above, except that the 2 mL sample solution was replaced by deionized water. The quantification was done based on a calibration curve obtained with glucose (linear range 1.2–12 μg/mL, R^2^ = 0.998).

### 3.5. HPLC–QTOF–MS/MS Analysis

The extracts of small-molecule saccharides were passed through 0.22 μm filter for HPLC–QTOF–MS/MS analysis. Chromatographic analysis was carried out on an Agilent 1200 liquid chromatography system coupled with QTOF–MS/MS, which was equipped with an electrospray interface (Agilent 6530, Agilent Technologies, Santa Clara, CA, USA), in accordance with our previous studies [37]. Various mixtures of water, methanol, and acetonitrile at flow rate 1.2 mL/min, 1.0 mL/min, 0.8 mL/min, 0.5 mL/min, and 0.3 mL/min were tested as the mobile phase. The detection temperature was tested at 15, 25, 35, and 45 °C. For most of the measurements, acetonitrile and ultrapure water were used as mobile phase A and B, respectively. Solvent flow rate was 1.0 mL/min and the column (Prevail Carbohydrate ES 5 μm, 250 mm × 4.6 mm, Alltech, New Westminster, Canada) was operated at 25 °C. The solvent gradient was used as follows: 75% A and 25% B. The condition of the QTOF was as follows: scan range 100–1500 *m*/*z*; drying gas (N_2_) flow rate, 8.0 L/min; drying gas temperature, 320 °C; sheath gas temperature, 320 °C; capillary voltage, 3.5 kV; fragmentor, 110 V; collision energy at 10, 15 and 20 eV. The operation and acquisition of data were controlled by Agilent Mass Hunter LC/MS Acquisition console, while the analysis of data was done by Qualitative Analysis B.05.00. Monosaccharides and oligosaccharides were determined according to precursor ions, the fragment ions and retention time comparing to those of the standard compounds.

### 3.6. Validation of the Methods for Quantitative Characterization of Monosaccharides and Oligosaccharides

Monosaccharides and oligosaccharides were quantitatively determined as described previously, but with minor modifications [38]. Briefly, standard solutions of monosaccharides and oligosaccharides at a series of appropriate concentrations were prepared for the construction of calibration curves, which were constructed by plotting the extracted ion chromatograms (EIC) peak area versus the concentration. The LOD and LOQ were calculated at approximately three-fold and ten-fold of the signal-to-noise (S/N) ratios, respectively. The measurement of the interday and intraday variabilities was used to determine the precision of this method. The standard solution was analyzed for six replicates within the same day, and additionally, on three consecutive days for evaluating intraday and interday variabilities, respectively. For the stability assessment, the sample extract was analyzed at 0, 2, 4, 8, 12, 24, and 48 h at room temperature. Recovery was determined by adding an accurately-measured amount of the standard compounds to the sample. Relative standard deviation (RSD) was used to assess the results.

### 3.7. PCA Statistical Analysis

The experiments were done in triplicate with three samples, and statistical analyses were performed using the statistical software R (https://www.r–project.org). A 30 × 11 matrix was constructed for the multivariate data treatment. The determined peak area value of the EIC responses of monosaccharides, oligosaccharides, polysaccharides, and saccharides derivatives were defined as variables, and were therefore placed in the columns. The thirty extracts were defined as samples and placed in rows. The data were imported by R software and treated using ggbiplot package to perform Principal Component Analysis (PCA) [39].

## 4. Conclusions

A sensitive and rapid analytical method was developed for the simultaneous identification and dynamic analysis of saccharide compounds during steam processing of *P. cyrtonema* using HPLC–QTOF–MS/MS. Fructose, sorbitol, glucose, galactose, sucrose, and 1-kestose were identified in the extract of the rhizomes of *P. cyrtonema*. Additionally, a large number of oligosaccharides constituted of fructose units through β-(2→1) or β-(2→6). Polysaccharides and oligosaccharides were decomposed to monosaccharides during repeated steaming processes, since the contents of glucose, galactose, and fructose increased, while those of sucrose, 1-kestose and polysaccharides decreased. Fructose was revealed to be the main determinant for the increasing sweetness after steaming of *P. cyrtonema*. Principal component analysis using saccharides as variables could substantially separate fresh and processed rhizomes of *P. cyrtonema* by repeated steaming 1–4 times, while samples with 4–9 times repeated steaming were grouped together. The samples gradually shifted along the PC1 (72.4%) axis with increases in the number of repeated times of steaming. The small-molecule saccharides, especially fructose, could be considered as markers for the steam process of rhizomes of *P. cyrtonema*.

## Figures and Tables

**Table 1:** Linear regression, LOD and LOQ of investigated compounds.

Compounds	Range (μg/mL)	Regression Equation ^a^	R^2^	LOD ^b^ (μg/mL)	LOQ ^c^ (μg/mL)
Fructose ^d^	6.4–64	y = 71886x + 682691	0.996	0.48	1.60
Glucose ^d^	15.2–152	y = 238502x + 2043001	0.991	0.90	2.99
Glucose ^e^	15.2–152	y = 457396x + 2818970	0.992	0.87	2.89
Galactose ^d^	15.7–157	y = 65662x + 479229	0.992	3.63	12.10
Sucrose ^d^	7.1–71	y = 296456x + 1182954	0.994	0.41	1.33
1-Kestose ^d^	73–366	y = 71886x + 922640	0.995	0.92	3.06

^a^ y: peak area, x: concentration of the analyte (μg/mL); ^b^ LOD, limit of detection; ^c^ LOQ, limit of quantification; ^d^ solvent flow rate at 1.0 mL/min; ^e^ solvent flow rate at 0.3 mL/min.

**Table 2:** Precision, stability and recovery of investigated compounds.

Compounds	Precision (*n* = 6)		Stability (48 h) (RSD, %)	Recovery (*n* = 3)	
	Intra-Day (RSD, %)	Inter-Day (RSD, %)		Mean (%)	RSD (%)
Fructose ^a^	1.37	1.82	2.29	96.7	2.37
Glucose ^a^	1.24	2.56	3.01	94.3	2.46
Glucose ^b^	1.62	2.29	1.48	98.3	2.13
Galactose ^a^	1.91	3.51	2.12	96.7	3.13
Sucrose ^a^	1.84	3.27	2.14	107.5	6.39
1-Kestose ^a^	0.93	2.22	1.29	96.1	3.71

^a^ solvent flow rate at 1.0 mL/min; ^b^ solvent flow rate at 0.3 mL/min.

**Table 3:** Identification of small–molecule saccharides by HPLC–QTOF–MS/MS technology

No	t_R_ (min)	[M − H]^−^ (*m*/*z*) (Δppm)	Fragment Ions (*m*/*z*)	Molecular Formula	Compound
1	7.09	323.1003 (−4.98)	323.0990; 179.0564; 161.0461; 143.0364; 113.0258; 101.0251	C_12_H_20_O_10_	Hexose derivative
2	8.16	323.1010 (−6.13)	323.1011; 179.0567; 161.0464; 143.0386; 113.0230; 101.0267	C_12_H_20_O_10_	Hexose derivative
3	9.12	179.0581 (−0.58)	179.0533; 161.0458; 143.0354; 113.0242; 101.0245	C_6_H_12_O_6_	Fructose
4	10.43	181.0739 (−0.36)	181.0715; 163.0629; 149.0436; 119.0338; 101.0247	C_6_H_14_O_6_	Sorbitol
5 ^a^	11.60	179.0566 (−2.94)	179.0583; 161.0433; 143.0350; 113.0248; 101.0248	C_6_H_12_O_6_	Glucose
6 ^a^	11.60	179.0585 (−0.08)	179.0574; 161.0480; 143.0336; 112.9853; 101.0246	C_6_H_12_O_6_	Galactose
7	14.4	179.0587 (−0.65)	179.0583; 161.0451; 143.0378; 112.9846; 101.0241	C_6_H_12_O_6_	Hexose
8	15.24	341.1125 (−1.14)	341.1090; 179.0555; 161.0465; 143.0338; 119.0350; 101.0243	C_12_H_22_O_11_	Sucrose
9	23.71	503.1666 (−1.1)	503.1668; 341.1101; 323.0988; 221.0670; 179.0554; 161.0441; 143.0328; 113.0220; 101.0240	C_18_H_32_O_16_	Trisaccharide
10	26.49	503.1670 (−4.19)	503.1668; 341.1054; 323.0986; 221.0655; 179.0553; 161.0467; 143.0368; 113.0255; 101.0244	C_18_H_32_O_16_	1-Kestose
11	33.15	503.1653 (2.62)	503.1653; 341.1031; 323.0936; 221.0616; 179.0513; 161.0412; 143.0308; 113.0216; 101.0219	C_18_H_32_O_16_	Raffinose

^a^ Characterization with sole standard glucose or galactose.

## Supplementary Materials

The Supplementary Materials are available online. Figure S1: Total ion chromatogram of fructose at different concentration (A) and nine times steam-treated samples of P. cyrtonema diluted to 20-fold and 100-fold (B) by HPLC–QTOF–MS/MS at solvent flow rate 1.0 mL/min.
