# Supplementary material for: Simultaneous Identification and Dynamic Analysis of Saccharides during Steam Processing of Rhizomes of Polygonatum cyrtonema by HPLC–QTOF–MS/MS
Source: Molecules. 2018 Nov 2;23(11):2855. doi: 10.3390/molecules23112855 (PMC6278431; doi:10.3390/molecules23112855)
Supplement: Supplementary file 1 [file molecules-23-02855-s001.pdf]

Supplementary material (Jin et al)

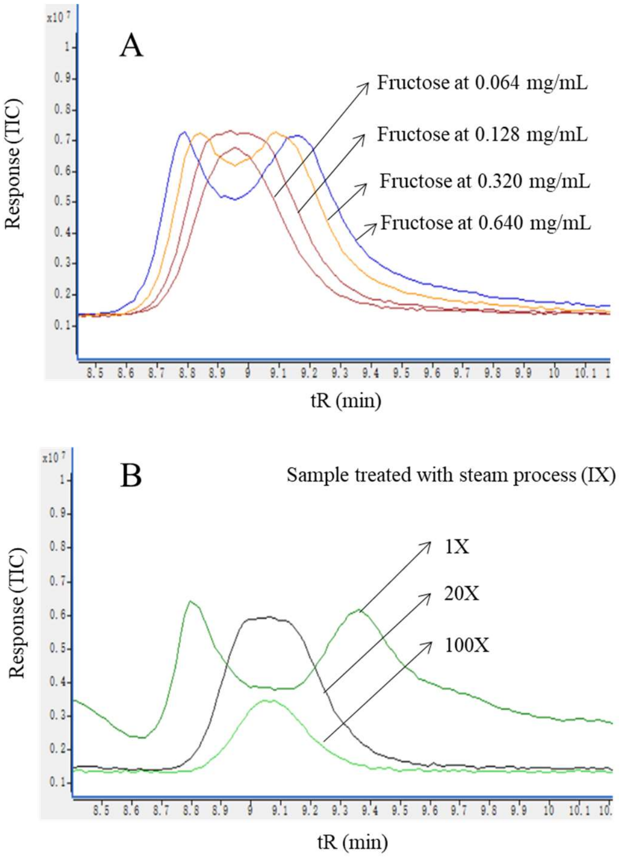

Figure S1. Total ion chromatogram of fructose at different concentration (A) and nine times steam-treated samples of *P. cyrtonema* diluted to 20–fold and 100–fold (B) by HPLC–QTOF–MS/MS at solvent flow rate 1.0 mL/min.
